# Virtual or in-person? a census of residency interview formats for match 2025

**DOI:** 10.1080/10872981.2026.2662719

**Published:** 2026-04-21

**Authors:** Katherine Velicki, Zoe VanderHoek, Ryan Christ, Brian Prigmore, Molly Joyce, Haley D. Smith, Ruth Carver Bondurant, Sydney Laxson, Elise Brannen, Fiona Stefanik, Jenny Rose Park, Lori Sun, Eden VanderHoek, Alexandra Hoffman, Derek Wong, Zachary Yellowman, Patrick Quinn, Kenneth Gundle

**Affiliations:** a Department of Orthopaedics and Rehabilitation at Oregon Health and Science University, Portland, Oregon, United States; b Oregon Health and Science University School of Medicine, Portland, Oregon, United States; c Department of Genetics at Yale University School of Medicine, New Haven, Connecticut, United States; d Orthopaedics Department at University of Utah Health, Salt Lake City, Utah, United States; e Oregon Health and Science University, Portland, Oregon, United States; f Department of Surgery at Oregon Health and Science University, Portland, Oregon, United States; g University of Wisconsin, Madison, Wisconsin, United States; h Medical College of Wisconsin, Milwaukee, Wisconsin, United States

**Keywords:** Residency interviews, virtual, in-person, climate change, medical school debt

## Abstract

**Introduction:**

Since the Match was established in 1952, residency interviews in the United States were conducted in person. This abruptly changed in May 2020, when the Coalition for Physician Accountability mandated that programs conduct interviews virtually for the 2020–2021 application season. While the Association of American Medical Colleges (AAMC) recommended virtual interviews in 2024, it is not known whether residency programs conducted virtual, hybrid, or in-person interviews in the application cycle that followed.

**Methods:**

A list of United States (US) residency programs from 23 specialties was compiled using the AAMC Electronic Residency Application Service (ERAS) Directory website. Interview format (virtual, hybrid, or in-person) for each program in the 2024–2025 application cycle was determined from correspondence with program coordinators, program websites, and specialty-specific databases. Pearson’s Chi-squared test was performed to determine whether there was a statistically significant difference in interview formats across specialties.

**Results:**

A total of 4863 residency programs across 23 specialties were found on the ERAS Directory. Researchers successfully determined interview formats for 4014 (82.5%) of these programs. The majority (2989, 74.5%) conducted virtual interviews, while others opted for in-person (700, 17.4%) and hybrid (325, 8.1%) interviews. In-person interviews were most favored by otolaryngology (86.3%), plastic surgery (85.2%), neurosurgery (84.5%), orthopaedic surgery (70.3%), and thoracic surgery (56.0%). Most programs from other specialties held virtual interviews. These inter-specialty differences were statistically significant (*p *< 2.2 x 10^−16^).

**Discussion:**

While most US residency programs conducted virtual interviews for Match 2025, many surgical subspecialties returned to in-person interviews. This census may inform stakeholders as they consider the future of residency interviews in years to come.

## Introduction

Since the establishment of the Match in 1952, interviews for residency in the United States (US) were traditionally conducted in person [1]. This abruptly changed in May 2020, when the Coalition for Physician Accountability mandated that all residency programmes conduct interviews virtually for the 2020-2021 application season [2]. In the years since the COVID−19 pandemic, residency programmes across the country have experimented with virtual, hybrid, and in-person interview structures.

The Association of American Medical Colleges (AAMC) published a formal recommendation supporting virtual interviews in June 2024 [3]. Applicant convenience, lower costs, and smaller carbon footprints were cited as some of the biggest advantages of the virtual format [4]. Nevertheless, the AAMC recommendation for virtual interviews in 2024 was not a mandate. Residency programme leaders were ultimately given the freedom to choose virtual, hybrid, or in-person interviews for the Match 2025 cycle. No group has published data on which interview formats they ultimately chose.

While programme leaders may be aware of changes within their own specialty, it is unclear if the decisions across fields are divergent. How are residency programmes currently conducting their interviews? A total of 28,760 US seniors applied for Match 2025. Each applicant attended a median of 14 interviews [5]. This time-honoured rite of passage in medical training affects many lives. We hope our data helps stakeholders better understand the current state of the residency interview process as they plan its future.

## Materials and methods

This study was structured as a census of residency interview formats for Match 2025 in the United States. We selected the following 23 residency specialties to focus on: anaesthesiology, dermatology, diagnostic radiology, emergency medicine, family medicine, general surgery, internal medicine, interventional radiology, neurology, neurosurgery, obstetrics and gynaecologist, orthopaedic surgery, otolaryngology, pathology, paediatrics, physical medicine and rehabilitation, plastic surgery, psychiatry, radiation oncology, thoracic surgery, urology, and vascular surgery. Our team then compiled lists of all residency programmes in each specialty from the AAMC Electronic Residency Application Service (ERAS) Directory website during the fall of 2024 [6]. Military programmes and those located in Puerto Rico were excluded.

Residency programme lists were divided among researchers for data collection. To determine programme interview formats, researchers were instructed to use correspondence with programme coordinators, programme websites, and specialty-specific databases as reliable sources of information. Databases were available from the following organisations: American Council of Educators in Plastic Surgery, Society of Academic Urologists, Society of Neurological Surgeons, Society for Academic Emergency Medicine, and American Orthopaedic Association. In rare cases when these sources were insufficient, researchers were permitted to use programme social media accounts and reports from contemporary applicants. Interview formats were coded by the researchers as virtual, hybrid, or in-person. Ophthalmology interviews were assumed to occur virtually given the Association of University Professors of Ophthalmology’s mandate for virtual interviews [7].

The outcome of this study was the percentages of United States residency programmes that conducted virtual, hybrid, and in-person interviews, respectively. Pearson’s Chi-squared test was used to determine whether interview format differences across specialties were statistically significant. The project was submitted to the Institutional Review Board and was exempted from approval due to lack of human subjects. The authors report there are no competing interests or funding sources to declare. The authors confirm that the data supporting the findings of this study are available within the article and/or its supplementary materials. Any reasonable requests for the data are available by request from the corresponding author.

## Results

A total of 4863 residency programmes from 23 specialties were identified in the AAMC ERAS Directory website. Of these, residency interview format was successfully determined in 4014 cases (82.5%), leaving 849 programmes unknown. Individual specialty completion rates ranging from 67.6% to 100% (Table 1).

**Table 1.** Percentage of known interview formats by specialty.

Specialty	Total programmes	Programmes with known interview format	Percentage known
Ophthalmology	125	Virtual mandate	100%
Otolaryngology	124	124	100%
Emergency medicine	280	277	98.9%
Plastic surgery	89	88	98.9%
Urology	144	142	98.6%
Neurosurgery	119	116	97.5%
Interventional radiology	99	93	93.9%
Orthopaedic surgery	198	182	91.9%
Paediatrics	206	179	86.9%
Neurology	183	153	83.6%
Family medicine	762	631	82.8%
Anaesthesiology	174	144	82.8%
Physical medicine & rehabilitation	110	90	81.8%
Radiation oncology	88	71	80.7%
Pathology	139	112	80.6%
Psychiatry	313	243	77.6%
Diagnostic radiology	190	145	76.3%
Vascular surgery	74	56	75.7%
Dermatology	139	104	74.8%
Obstetrics & gynaecologist	288	213	74.0%
Internal medicine	628	453	72.1%
General surgery	354	248	70.1%
Thoracic surgery	37	25	67.6%

Ophthalmology interviews were assumed to occur virtually (125 programmes, 3.1%). Otherwise, the most common sources used to determine interview format were email and phone correspondence with programme coordinators (1816 programmes, 45.2%), programme websites (1345 programmes, 33.5%) and specialty-specific databases (586 programmes, 14.6%). Social media posts and accounts from current applicants were rarely needed (142 programmes, 3.5%).

**Figure 1.**
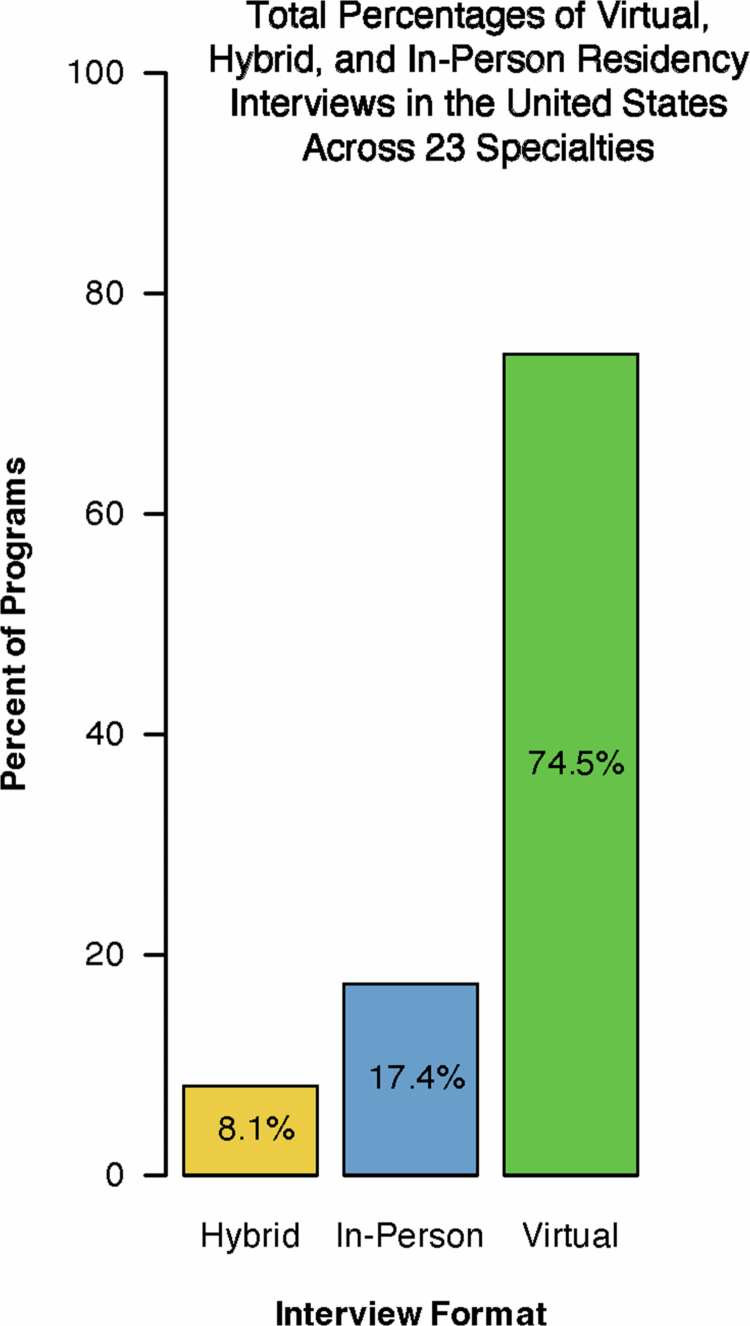
Total percentages of virtual, hybrid, and in-person residency interviews in the United States across 23 specialties.

**Figure 2.**
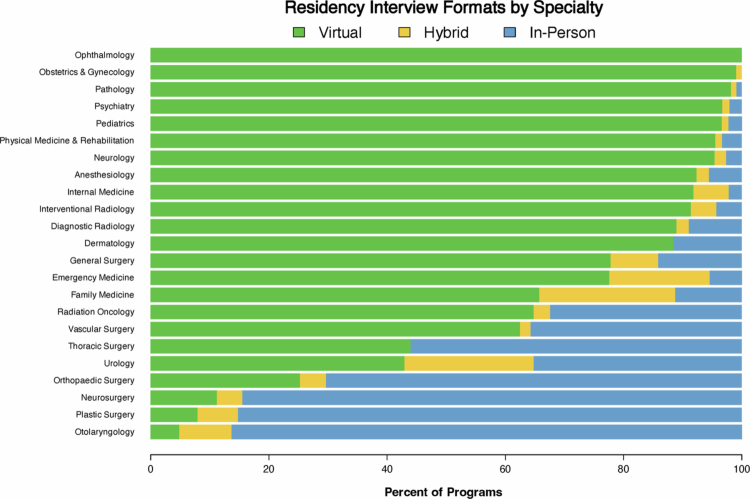
Residency interview formats by specialty.

As shown in Figure 1, most residency programmes interviewed virtually (74.5%) while some conducted via in-person (700 programmes, 17.4%) and hybrid (325 programmes, 8.1%) interviews. Choice of interview format varied substantially between specialties (Figure 2). These inter-specialty differences were statistically significant (*p* < 2.2 × 10^−16^). In-person interviews were most favoured by otolaryngology (86.3%), plastic surgery (85.2%), neurosurgery (84.5%), orthopaedic surgery (70.3%), and thoracic surgery (56.0%).

## Discussion

This study reveals substantial variation in residency interview formats between specialties for Match 2025. Most programmes continued virtual interviews. At a time when 70% of medical students carry educational debt and federal loans are being capped, virtual interviews reduce the financial burden of medical training [8,9]. Virtual interviews also have an environmental advantage. Using Hampshire et al.’s estimate of 4.31 tons of carbon dioxide (CO2) per applicant [10], the 28,760 US seniors that participated in Match 2025 would have burned the equivalent of about 12.6 million gallons of gasoline in a fully in-person interview cycle [5,11].

Despite these benefits, many surgical subspecialties returned to in-person interviews. Leaders in these fields have cited difficulty assessing applicant “fit”, personality, communication skills, and commitment to the specialty using the virtual format [12]. Some also have noted that in-person interviews allow candidates to experience a programme’s location and culture [13–15].

Given these valid arguments, perhaps the most optimal future of residency interviewing is hybrid [16,17]. In Match 2025, most US seniors (73% MD, 76% DO) matched into one of their top 3 ranked residency programmes [18]. What if applicants interviewed in person at their top three choices and virtually for the rest? This, combined with programme caps of 3 in-person interviews per residency slot, could ensure that applicants are investing their time, money, and carbon in residencies where they have a high likelihood of matching.

Our study has weaknesses. Multiple researchers collected data from multiple sources to determine interview formats, introducing risk of information bias. Opinions and motivations behind interview format decisions were not explored. Nevertheless, we hope that this relatively complete and recent census informs stakeholders as they consider the future of residency interviews in years to come.

## Data Availability

The data that support the findings of this study are available on request from the corresponding author upon reasonable request.
